# Predicting habitat suitability for *Ixodes* *ricinus* and *Ixodes* *persulcatus* ticks in Finland

**DOI:** 10.1186/s13071-022-05410-8

**Published:** 2022-08-30

**Authors:** Ruut Uusitalo, Mika Siljander, Andreas Lindén, Jani J. Sormunen, Juha Aalto, Guy Hendrickx, Eva Kallio, Andrea Vajda, Hilppa Gregow, Heikki Henttonen, Cedric Marsboom, Essi M. Korhonen, Tarja Sironen, Petri Pellikka, Olli Vapalahti

**Affiliations:** 1 Department of Geosciences and Geography, University of Helsinki, P.O. Box 64, 00014 Helsinki, Finland; 2 Department of Virology, University of Helsinki, P.O. Box 21, 00014 Helsinki, Finland; 3 Department of Veterinary Biosciences, University of Helsinki, P.O. Box 66, 00014 Helsinki, Finland; 4 Natural Resources Institute Finland, P.O. Box 2, 00791 Helsinki, Finland; 5 Biodiversity Unit, University of Turku, 20014 Turku, Finland; 6 Department of Biology, University of Turku, 20014 Turku, Finland; 7 Weather and Climate Change Impact Research Unit, Finnish Meteorological Institute, P.O. Box 503, 00101 Helsinki, Finland; 8 Research Department, AVIA-GIS, Zoersel, Belgium; 9 Department of Biological and Environmental Science and School of Resource Wisdom, University of Jyväskylä, 40014 Jyväskylä, Finland; 10 Virology and Immunology, HUSLAB, Helsinki University Hospital, Helsinki, Finland; 11 Helsinki Institute of Sustainability Science, University of Helsinki, Helsinki, Finland; 12 Institute for Atmospheric and Earth System Research, University of Helsinki, Helsinki, Finland

**Keywords:** *Ixodesricinus*, *Ixodespersulcatus*, Species distribution modelling, Ensemble prediction, Tick-borne pathogen, *Borreliaburgdorferi* sensu lato

## Abstract

**Background:**

Ticks are responsible for transmitting several notable pathogens worldwide. Finland lies in a zone where two human-biting tick species co-occur: *Ixodes* *ricinus* and *Ixodes* *persulcatus*. Tick densities have increased in boreal regions worldwide during past decades, and tick-borne pathogens have been identified as one of the major threats to public health in the face of climate change.

**Methods:**

We used species distribution modelling techniques to predict the distributions of *I.* *ricinus* and *I.* *persulcatus,* using aggregated historical data from 2014 to 2020 and new tick occurrence data from 2021. By aiming to fill the gaps in tick occurrence data, we created a new sampling strategy across Finland. We also screened for tick-borne encephalitis virus (TBEV) and *Borrelia* from the newly collected ticks. Climate, land use and vegetation data, and population densities of the tick hosts were used in various combinations on four data sets to estimate tick species’ distributions across mainland Finland with a 1-km resolution.

**Results:**

In the 2021 survey, 89 new locations were sampled of which 25 new presences and 63 absences were found for *I.* *ricinus* and one new presence and 88 absences for *I.* *persulcatus*. A total of 502 ticks were collected and analysed; no ticks were positive for TBEV, while 56 (47%) of the 120 pools, including adult, nymph, and larva pools, were positive for *Borrelia* (minimum infection rate 11.2%, respectively). Our prediction results demonstrate that two combined predictor data sets based on ensemble mean models yielded the highest predictive accuracy for both *I.* *ricinus* (AUC = 0.91, 0.94) and *I.* *persulcatus* (AUC = 0.93, 0.96). The suitable habitats for *I.* *ricinus* were determined by higher relative humidity, air temperature, precipitation sum, and middle-infrared reflectance levels and higher densities of white-tailed deer, European hare, and red fox. For *I.* *persulcatus*, locations with greater precipitation and air temperature and higher white-tailed deer, roe deer, and mountain hare densities were associated with higher occurrence probabilities. Suitable habitats for *I.* *ricinus* ranged from southern Finland up to Central Ostrobothnia and North Karelia, excluding areas in Ostrobothnia and Pirkanmaa. For *I.* *persulcatus*, suitable areas were located along the western coast from Ostrobothnia to southern Lapland, in North Karelia, North Savo, Kainuu, and areas in Pirkanmaa and Päijät-Häme.

**Conclusions:**

This is the first study conducted in Finland that estimates potential tick species distributions using environmental and host data. Our results can be utilized in vector control strategies, as supporting material in recommendations issued by public health authorities, and as predictor data for modelling the risk for tick-borne diseases.

**Supplementary Information:**

The online version contains supplementary material available at 10.1186/s13071-022-05410-8.

## Background

In temperate and boreal forests, ticks are among the most important arthropods transmitting viruses and other pathogens causing diseases in humans. In Northern Europe, among the most prevalent tick-borne pathogens are *Borrelia* *burgdorferi* (s.l.) bacteria causing Lyme borreliosis (LB) and tick-borne encephalitis virus (TBEV) causing tick-borne encephalitis (TBE). The abundances and distributions of vectors and their hosts are mainly restricted by their habitat suitability, determined largely by climatic and environmental conditions, similarly to other arthropods. Finland lies in a zone with overlapping geographical distributions of two tick species, the castor bean tick *Ixodes* *ricinus* Linnaeus 1758 and the taiga tick *Ixodes* *persulcatus* Schulze 1930. Tick abundances have increased in Finland, which has been mirrored by a rising incidence of tick-borne diseases (TBDs) in the country during the past decades [1–4]. The distribution of *I.* *ricinus* extends throughout Europe from Sweden to North Africa and from Ireland to the Urals [5, 6], and the distribution of *I.* *persulcatus* extends from Fennoscandia to Japan. During the last 2 decades, the taiga tick, *I.* *persulcatus*, has spread to new areas in Northern Europe: from eastern Finland to the north-western coast of Finland and all the way to eastern Sweden [7–9]. Currently, ticks occur throughout Finland, excluding most of Lapland [9]. *Ixodes* *ricinus* is predominant in southern Finland while *I.* *persulcatus* prevails in the eastern regions of Finland and on the north-western coast up to southern Lapland, although *I.* *persulcatus* may be spreading southwards, with recent records from the capital region along the south coast [10]. Central Finland is considered a sympatric area where both species occur.

Knowledge of tick seasonalities, i.e. the time periods when ticks are active, is important because of its public health relevance, but also for understanding how environmental and other factors influence their distributions. *Ixodes* *ricinus* is active from May to September in Finland, with two activity peaks. Adult *I.* *ricinus* activity peaks in July–August and nymph activity peaks in September [9, 11], while *I.* *ricinus* nymphs and adult females have been found to peak in May–June [12] and in August–September [12, 13]. The seasonal activity of *I.* *persulcatus* adults in Finland is unimodal, as they are active in May–July, with the highest activity peak in May [9, 11, 14]. Tick reproduction and abundance and pathogen transmission are dependent on the presence of a suitable vertebrate host. Adult and nymphal *I.* *ricinus* and *I.* *persulcatus* often feed on small to medium-sized animals, such as rodents, birds, foxes, and hares, but also on large-sized animals including deer, moose, and humans [15–17]. In contrast to *I.* *ricinus* nymphs, *I.* *persulcatus* nymphs rarely feed on larger hosts, including humans [14]. Larval *I.* *ricinus* and *I.* *persulcatus* feed mainly on terrestrial vertebrates, such as voles or birds, as they are numerous and move close to the ground in the vicinity of tick larvae [18, 19]. Tick densities vary largely depending on geographical location. Generally, 100–1000/100 m^2^ is considered high density for *I.* *ricinus* nymphs in Central Europe, while 10–13/100 m^2^ and 10/100 m^2^ already indicate high densities for *I.* *ricinus* [2, 20] and *I.* *persulcatus* nymphs or adults in Finland [14], respectively. High density numbers in Finland coincide with those observed for *I.* *persulcatus* in northern Sweden [21].

Weather, climate change, and the environment influence habitat suitability, vector activity, and the rate of vector development [22, 23]. Ticks need sufficiently high temperatures to complete their development [24]. *Ixodes* *ricinus* and *I.* *persulcatus* differ in terms of their temperature-related activity, with *I.* *persulcatus* being active at lower temperatures [25–27]. Generally, warm temperatures and increased rainfall positively affect tick densities [22], but extremely high temperatures combined with decreased rainfall may reduce tick populations [28]. Water vapour is the main source of moisture for active unfed ticks, and it is only absorbed at sufficiently high relative air humidity [24]. *Ixodes* *ricinus* requires high relative humidity (> 80%) to survive during off-host periods [29]. In the northern distribution limit of the species, relative humidity was found to positively correlate with the abundances of *I.* *ricinus* nymphs and adults [2]. Saturation deficit was also found to positively influence the abundance of questing *I.* *ricinus* larvae and to positively influence adult abundance until the optimal value (3.16 mmHg) [12]. Despite less research conducted on *I.* *persulcatus* than on *I.* *ricinus* in their northern distribution limits, findings suggest that the climatic requirements of *I.* *persulcatus* differ from those of *I.* *ricinus.* *Ixodes* *persulcatus* occurs in areas where growing season length varies between 140 and 150 days, while *I.* *ricinus* prefer areas with a growing season of > 180 days [8]. Furthermore, *I.* *persulcatus* occurs in areas with less precipitation and a lower humidity index and temperature sum than the northern distribution limits of *I.* *ricinus* [8, 24]. Snow cover influences tick development, acting as an insulator that protects the nymphal ticks and larvae from freezing [30].

Species distribution modelling (SDM) methods can be used to determine the habitat suitability areas of the two species and to indicate the most influential factors affecting their distributions [31]. SDM techniques have widely been used to study *I.* *ricinus* occurrences [32–37] in Europe. In addition to studies focused on modelling *I.* *ricinus* occurrences, multiple studies have modelled *I.* *ricinus* densities [38–41], which requires more comprehensive and continuous tick sampling than needed for presence-absence modelling. No earlier SDM studies have estimated *I.* *persulcatus* distributions in Europe or Asia, although a few studies have estimated their probable occurrences [42, 43]. Earlier research in Finland on tick distributions shows occurrence point patterns [9] and *I.* *ricinus* [11, 12, 20] and *I.* *persulcatus* [11, 14] abundances at smaller scales, but SDM methods have not been conducted previously. Host data are difficult to obtain and are thus missing from the majority of SDM studies examining ticks. However, several statistical analyses on *I.* *ricinus* distributions have included tick host data [44–47].

In this study, we aim to (1) analyse the ticks collected in 2021 for species and possible pathogens: TBEV and *Borrelia*, (2) estimate the potential distributions of *I.* *ricinus* and *I.* *persulcatus* in mainland Finland and assess the model performance using various variable compositions, and (3) identify the most influential factors driving the spatial patterns. The study aim is to predict tick species occurrences instead of tick densities, and thus habitat suitability in the Results refers to the probability of species presence instead of the estimated abundance. Our study is based on data obtained from historical tick data sets from 2014 to 2020 and on new tick collections from 2021.

## Methods

### Study area

**Fig. 1:**
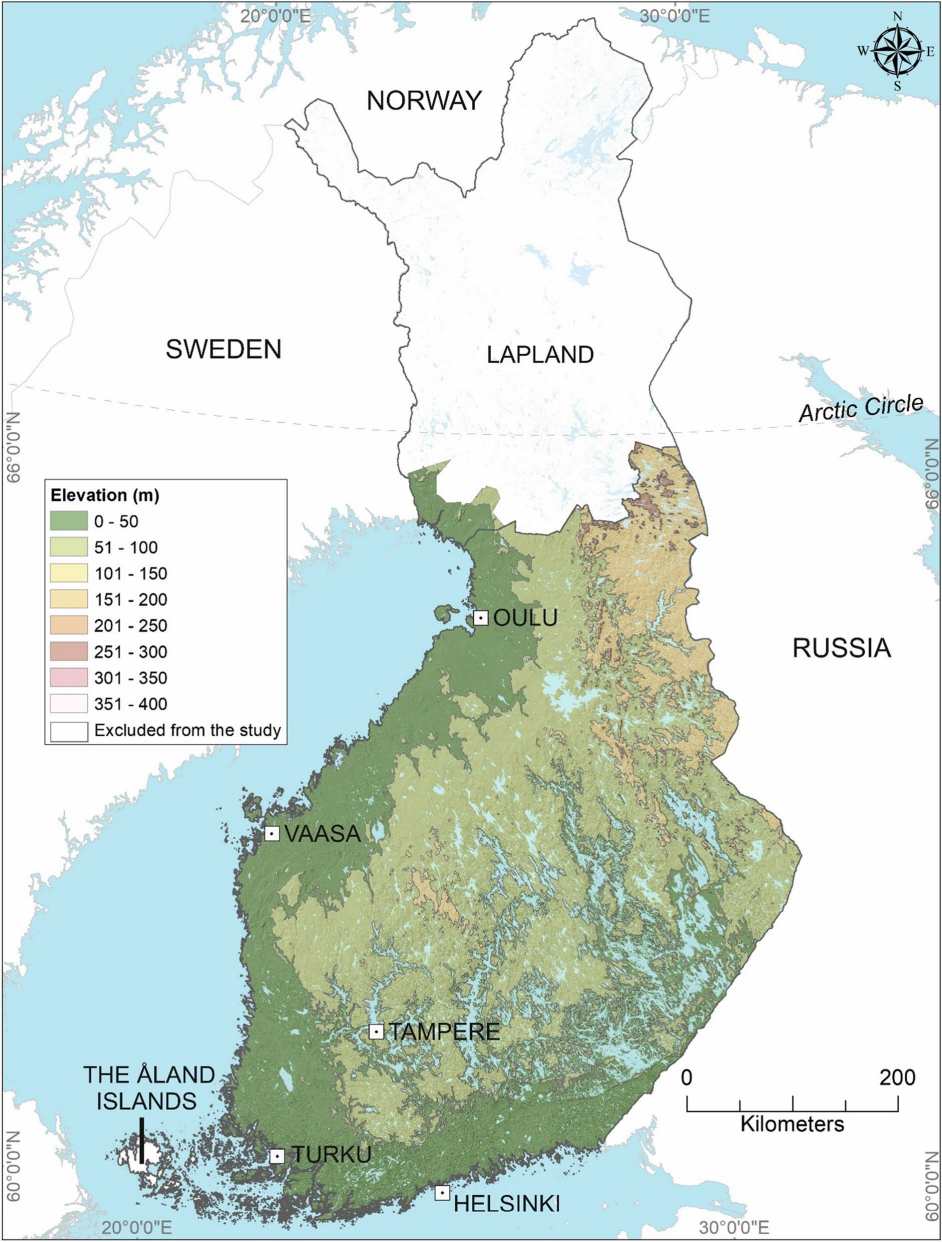
A map presenting the study area with elevation levels

Finland (59°50′N, 20°38′E, 70°09′N, 31°30′E) is located in Northern Europe between Sweden and Russia (Fig. 1). This study covers Finland, excluding the Åland Islands and most parts of Lapland. Only the south-western municipalities of Lapland were included, Simo, Keminmaa, Tornio, and Kemi, because of the presence of both tick species in the region [9].

### Tick data

#### Historical tick data

Historical tick data were collected by the Universities of Turku, Jyväskylä, and Helsinki. The majority of the data consisted of national crowdsourcing data, where citizens collected ticks across Finland from April to November, 2015, and sent them to the University of Turku for identification and further analysis [9]. The coordinates for the crowdsourcing data were manually gathered and recorded by biologists from the University of Turku, based on information provided in the letters used to send the ticks, along with their own assessment of suitable areas for ticks (based on topographic and satellite imagery). As for pinpointing the location where each tick got on its vessel, most ticks were sent in by dog owners after being removed from the dogs. These dog owners reported consistently walking approximately the same routes with their pets, and thus we would expect the ticks to have gotten on the dogs within a 500-m diameter of the mentioned walking areas. These factors naturally lead to uncertainty in the correctness of the coordinate points and, more notably, to uncertainty regarding the accuracy to which the coordinates depict the exact location for acquiring the tick. However, we would expect the coordinate points and surrounding environments to represent the tick acquisition area at a 1-km resolution in most cases. In addition, tick collections at smaller scales were conducted by the Universities of Helsinki, Turku, and Jyväskylä from 2014 to 2020. Aggregated historical data consisted of 4152 presences for *I.* *ricinus* and 986 presences for *I.* *persulcatus*.

#### Sampling strategy for tick collections in 2021

Historical *I.* *ricinus* data covered the entire study area well, but *I.* *persulcatus* occurrence data were spatially clustered. We therefore targeted the activity season of *I.* *persulcatus* from May to June in 2021 to fill the gaps in environmental space covered by the data. We created a GIS-based sampling strategy using both ESRI ArcGIS (version 10.3.1) (ESRI, Redlands, CA, USA) and VECMAP^®^ software [48] with the following criteria (Additional file 1: Fig. S1a). We created "regulated areas" as buffer zones around existing *I.* *persulcatus* occurrences within a 5-km radius to exclude them from the sampling strategy. Then, we created 500-m buffer zones around the roads to ensure accessibility. We used the CORINE Land cover CLC2018 data set available for Finland [49] to extract land cover classes known to be suitable habitats for ticks: a forest category including deciduous, coniferous, and mixed forests and a meadow category including sparsely wooded areas. We used subdivisions of landscape provinces to distinguish areas already well covered with *I.* *persulcatus* occurrences from four larger areas where further collections were needed. For each of the four areas, a random sample of 25 collections points (a total of 100 locations) was created in VECMAP^®^ based on the relative shares of the two land cover categories in the area (approximately 86% forests and 14% meadows). As ecotones are locations with most tick abundance [50], each created collection point was moved to the closest ecotone using a Google Maps Satellite map.

#### Tick collections and processing

Additional sampling of 100 locations was carried out from the beginning of May to the end of June 2021, which is considered the activity season of *I.* *persulcatus* (peak in May) in Finland [9, 11, 14]. Ticks were collected by slowly dragging a 1.0 × 1.5-m or a 1.0 × 1.0-m cotton cloth over 10-m sections, with a total of 400-m dragging sessions in each locality. This design was defined together with Finnish tick specialists. If ticks were not detected after 400 m of dragging, the site was considered an absence location. Sampling was conducted during the day or night but not on rainy days. Sampling date or time may affect the sampling results. All ticks attached to the cloth were collected using tweezers. The larvae, nymphs, and adults were separated, placed into 15-ml Greiner tubes with a grass stalk inserted in each tube, and transported to the Department of Virology at the University of Helsinki. Ticks were stored alive at 4 °C until homogenization. Ticks were pooled according to species, development stage, and collection site (adults 1; nymphs 1–5; larvae 1–50). RNA was extracted using a QIAamp Viral RNA Mini Kit (Qiagen) and DNA using a DNeasy Blood and Tissue DNA extraction kit (Qiagen, Hilden, Germany) according to the manufacturers’ instructions. Tick species was confirmed by molecular identification, using a species-specific duplex TaqMan real-time PCR assay, as previously described [1]. TBEV was screened using a quantitative real-time PCR assay [51], with the following modifications; assays were carried out in 20 μl of reaction volume, including 5 μl Taqman fast virus 1-step mastermix (Thermo Fischer Scientific), 0.4 μl forward primer, 0.6 μl reverse primer, 0.16 μl probe, 8.84 μl H_2_O, and 5 μl RNA. The thermal cycling profile was 50 °C for 5 min, followed by 20 s at 95 °C, prior to amplification (95 °C 15 s, 60 °C 30 s, 40 cycles). For *Borrelia*, we used a quantitative real-time PCR assay, as previously described [9]. All PCR reactions were performed on a T100™ Thermal Cycler (Bio-Rad, Germany).

### Data pre-processing

**Fig. 2:**
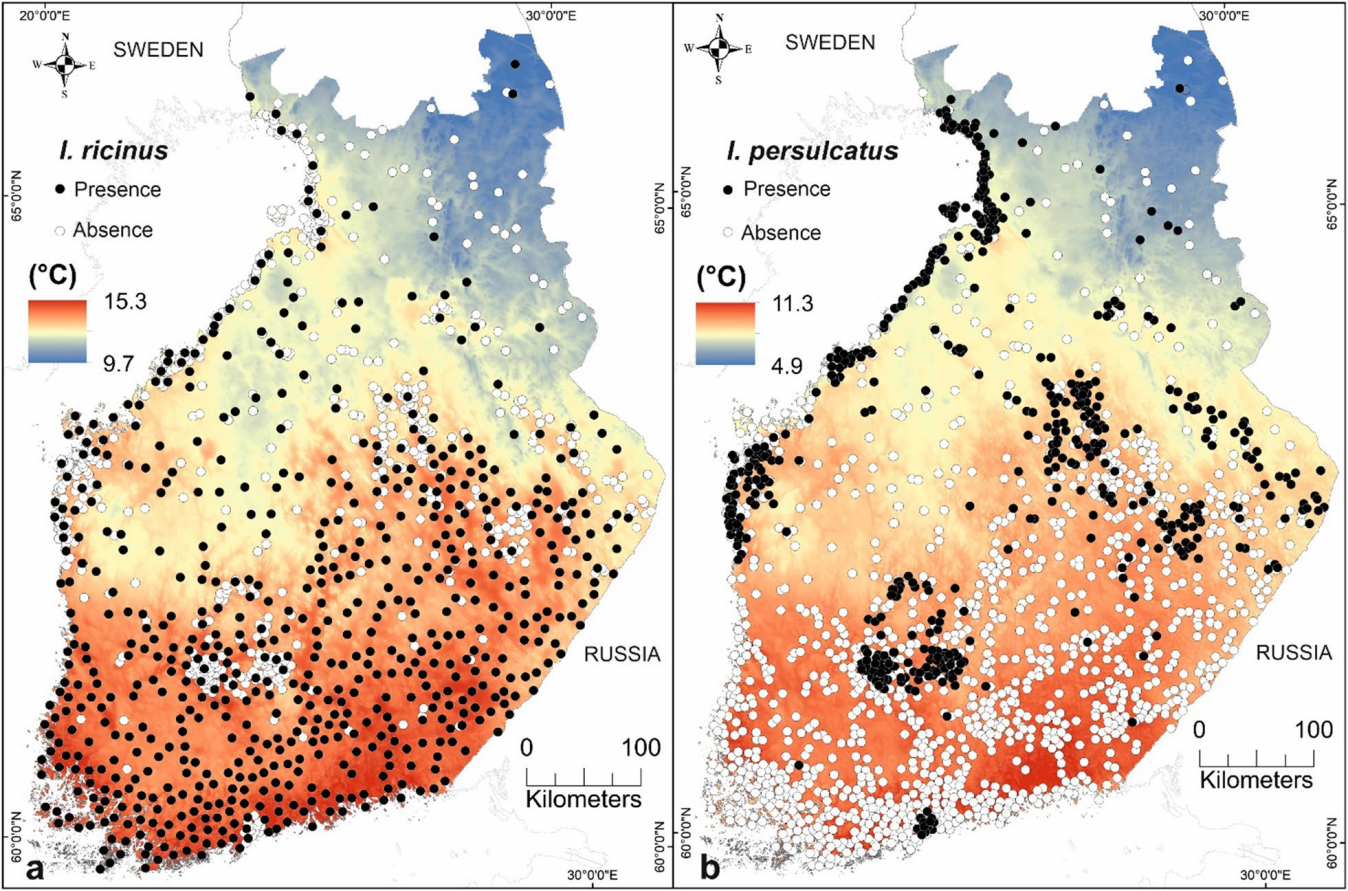
Presence–absence data of **a** *I.* *ricinus* and **b** *I.* *persulcatus* after data thinning, presented together with mean air temperature during the activity seasons of each species: May–September for *I.* *ricinus* and April–June for *I.* *persulcatus* in 2014–2021

Spatial autocorrelation (SA) refers to data or residuals correlated with themselves rather than being independent [52], and it may inflate the effective sample size and bias parameter estimates. To reduce SA, tick occurrence data were thinned using R package *Wallace* [53], which uses the *spThin* approach [54]. For *I.* *ricinus* presence-absence data, we used 10 km of spatial thinning for *I.* *ricinus* and 3 km of spatial thinning for *I.* *persulcatus*, which were considered *I.* *ricinus* absences with true absences (*N* = 88) obtained from the summer sampling in 2021. For *I.* *persulcatus* presence-absence data, 3 km of spatial thinning for *I.* *persulcatus* was used to obtain the highest possible number of presences and 5 km of spatial thinning for *I.* *ricinus.* These data were aggregated with data of true absences described above. After data thinning, *I.* *ricinus* data consisted of 622 presences and 637 absences, and *I.* *persulcatus* data consisted of 509 presences and 1289 absences (Fig. 2a, b).

### Geospatial data

**Fig. 3:**
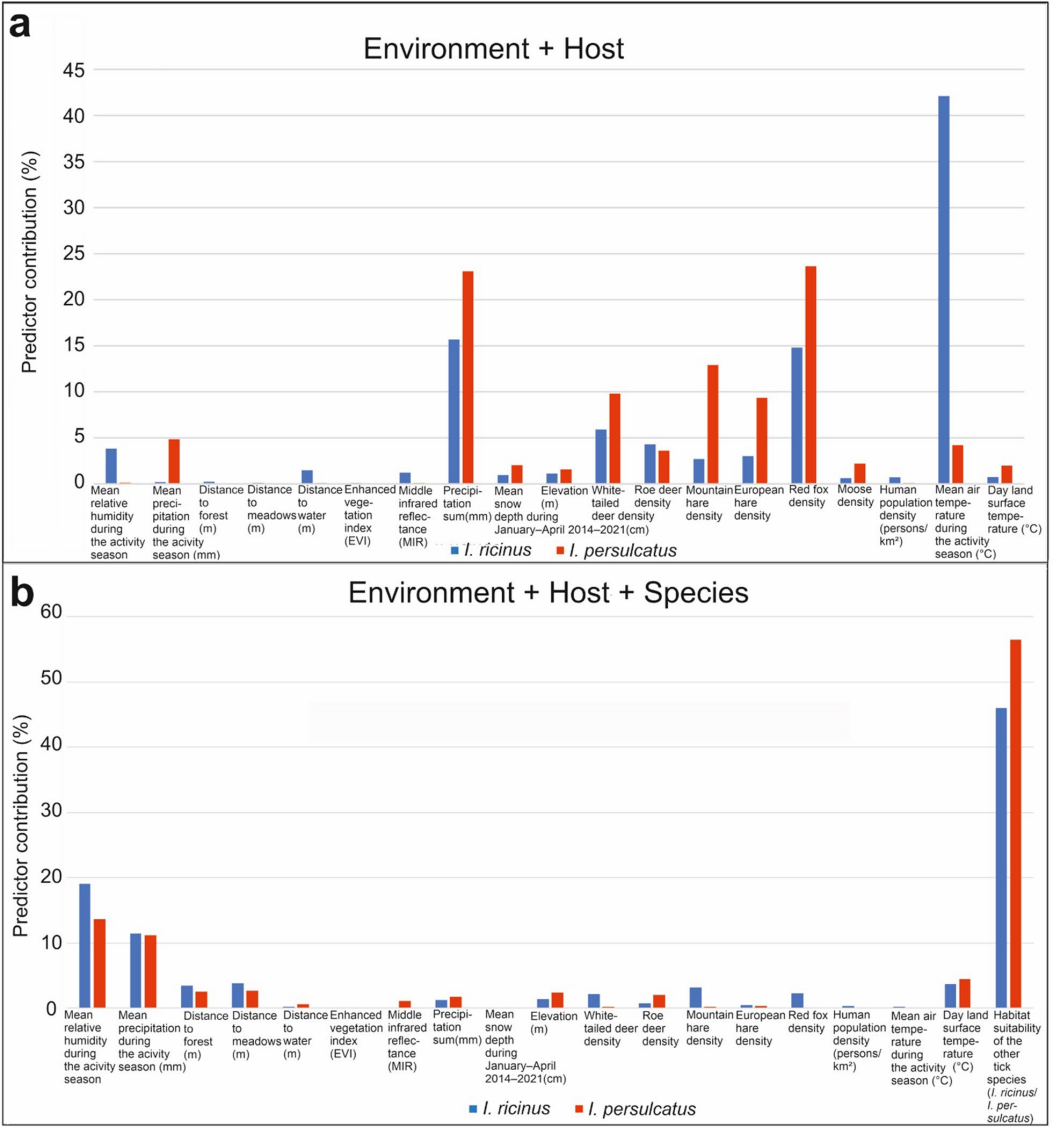
The relative contributions of the explanatory variables in the data set of **a** the environment and host and **b** the environment, host, and habitat suitability for the other species (*I.* *ricinus*/*I.* *persulcatus*) based on the mean ensemble models

Environmental, climate, and other predictors were selected based on factors known to influence the *I.* *ricinus* and *I.* *persulcatus* distributions. Environmental data for Finland were obtained from various sources and included data derived from satellite imagery, GIS layers, or interpolated data. Details of the predictor data are provided in Table 1. The initial data sets for both species included 25 predictors before running a multicollinearity analysis. Final predictors in each variable combination are seen in Fig. 3 and Additional file 5: Table S1. For monthly climate data (relative air humidity, air temperature, and precipitation), we calculated the mean for the activity seasons for both species: May–September for *I.* *ricinus* and April–June for *I.* *persulcatus*.

**Table 1:** Descriptions of geospatial data used in the study

Data layer(s)	Modifications	Year	Spatial resolution	References
Mean monthly air temperature (°C)	Calculated mean monthly air temperature during the activity season of *I.* *ricinus* (May–September) and *I.* *persulcatus* (April–June) in 2014–2021	2014–2021	1000 m	FMI [55]
Mean monthly precipitation (mm)	Calculated mean monthly precipitation during the activity season of *I.* *ricinus* (May–September) and *I.* *persulcatus* (April–June) in 2014–2021	2014–2021	1000 m	FMI [55]
Mean monthly snow depth (cm)	Calculated mean monthly snow depth in January–April 2014–2021	2014–2021	1000 m	FMI [55]
Mean precipitation during the growing season (mm)	Calculated mean precipitation during the growing season	Averages for 1981–2010	1000 m	FMI [56]
Mean heat summation during the growing season (°C day)	Calculated mean heat summation during the growing season	Averages for 1981–2010	1000 m	FMI [56]
Growing season length (GLS) (days)	Calculated growing season length (GLS)	Averages for 1981–2010	1000 m	FMI [56]
CORINE land cover 2018	Euclidean distances to water, meadow, and forest from tick species presence–absence points were calculated in ArcGIS	2018	20 m	SYKE [49]
Human population density (persons/km^2^)	Calculated as a sum	2019	1000 m	Statistics Finland [57]
Digital elevation model (DEM) (m)	Calculated mean elevation	2019	10 × 10 m	NLS of Finland [58]
Normalized difference vegetation index (NDVI)	Mean NDVI	2012–2020	1000 m	Global VIIRS data [59]
Enhanced vegetation index (EVI)	Mean EVI	2012–2020	1000 m	Global VIIRS data [59]
Land surface temperature (LST) (°C) Middle-infrared Reflectance (MIR)	Mean day and night LST Mean MIR	2012–2020 2012–2020	1000 m 1000 m	Global VIIRS data [59] Global VIIRS data [59]
Abundance indexes of red fox (*Vulpes* *vulpes*), roe deer (*Capreolus* *capreolus*), European hare (*Lepus* *europaeus)*, mountain hare (*Lepus* *timidus*), moose (*Alces* *alces*), and white-tailed deer (*Odocoileus* *virginianus*) Habitat suitability data of *I.* *ricinus/* *I.* *persulcatus*	Average snow track densities in a 50-km radius. Annual average values were averages over 2014–2021. Based on wildlife triangle census Habitat suitabilities for *I.* *ricinus* and *I.* *persulcatus* were estimated based on environmental and host data	2014–2021 Estimations based on 2014–2021 data	1000 m 1000 m	LUKE [60] Produced in this study

*FMI* Finnish Meteorological Institute, *SYKE* Finnish Environment Institute, *ESA* European Satellite Agency, *VIIRS* Global Visible Infrared Imaging Radiometer Suite, *LUKE* Natural Resources Institute Finland

### Host data

Host abundance estimates were based on snow track counts from the wildlife triangle census, which is a national monitoring scheme coordinated by the Natural Resources Institute Finland (LUKE). Abundance was approximated using a snow track index (*I* = tracks/10 km/day) for each species and triangle in an area, i.e., the number of snow tracks observed per 10 km of counted transect, further divided by the number of days during which snow tracks have accumulated (days since the last snowfall or since a round of “pre-mapping”). For each species occurrence, we calculated the average annual snow track index within a 50-km radius, and the annual indexes for that area were further averaged over the study period (2014–2021). The layer of species-specific distributions was formed by repeating this procedure over a 1 × 1-km grid (Table 1). The data consist of calculated average abundance indexes for European hare (*Lepus* *europaeus)*, mountain hare (*Lepus* *timidus*), moose (*Alces* *alces*), red fox (*Vulpes* *vulpes*), roe deer (*Capreolus* *capreolus*), and white-tailed deer (*Odocoileus* *virginianus*).

### Data preparation and data analysis

We used four variable compositions for the SDM predictions: environment only, host only, combined data on environmental and host variables, and combined data on environmental and host variables with suitability data of either tick species. In the fourth variable composition, the suitability data of either tick species were produced based on environmental and host data to reveal any potential effects of *I.* *ricinus* occurrence to *I.* *persulcatus* presence and vice versa. We decided to include this predictor because of field observations in which the one species has not been found in the vicinity of the other species. We used the biomod2 platform in R (version 3.4.6) [61, 62] to create SDMs to identify areas with suitable habitat conditions for *I.* *ricinus* and *I.* *persulcatus*. All geospatial data sets, including environmental and other data, were processed in ESRI ArcGIS (version 10.3.1) (ESRI, Redlands, CA, USA) and were set to the same spatial extent, geographic coordinate system (EUREF FIN TM35FIN, epsg:3067), and resolution (1 × 1 km). Multicollinearity of the variables was investigated using Variance Inflation Factors (VIFs), as implemented in R package *usdm* [63, 64]. Predictor VIFs were calculated and correlated variables were excluded in a stepwise procedure using a commonly applied threshold value of 10 [65, 66]. The following eight predictive modelling techniques were employed: generalized linear models (GLM) [67], generalized additive models (GAM) [68], classification tree analysis (CTA) [69], artificial neural networks (ANN) [70], multivariate adaptive regression splines (MARS) [71], generalized boosting models (GBM) [72], random forest (RF) [73], and maximum entropy (MAXENT) [74]. Flexible discriminant analysis (FDA) and surface range envelope (SRE) were excluded due to generally poor predictive performance [75, 76]. Models were mostly run using the default settings of biomod2, with the following exception: we used the GAM function in the mgcv package, with *k* = 3 as the basis dimension for the thin plate smoothing terms [77]. We used a cross-validation technique where we split the thinned data set into two subsets, one to calibrate the models (70%) and another to evaluate the models (30%) [31]. We repeated the calibration and evaluation sets 50 times for each model (400 model evaluation runs in total) [31]. To reduce the uncertainty related to the choice of a single modelling technique, we built ensemble predictions using the ensemble mean method that averages predictions across the best-performing individual models with the selected threshold (0.7 < AUC < 1.0) [62].

### The accuracy assessment

Sensitivity (the proportion of correctly predicted presences) and specificity (the proportion of correctly predicted absences) were calculated to quantify omission errors [78]. Sensitivity is calculated by dividing true presences (TP) by the sum of TP and false absences (FA). Similarly, specificity is calculated by dividing true absences (TA) by the sum of TA and false positives (FP). The area under the curve (AUC), used here to assess model performance, is the measure of a model’s ability to distinguish between these presence and absence classes. The AUC scores range from 0 to 1, with 0.5 being the threshold for predictions better than random [78] and > 0.7 being an acceptable threshold for predictions [79]. The variable importance of predictors, based on decreasing accuracy, was extracted from the biomod2 output [56]. Each importance value was normalized by dividing the value by the total sum of importance values to compare the most powerful variables. Partial dependency plots were generated to show the predictors’ estimated effects on tick distributions. Prediction uncertainty was assessed by the coefficient of variation (CV) of predictions, with a high CV value indicating high uncertainty in the predicted distributions [56].

## Results

### Additional tick collections in 2021

We identified the tick species from the additional data collections in 2021, and the ticks were screened for TBEV and *Borrelia* pathogens. We sampled 89 new locations, which included 25 new presences and 63 absences for *I.* *ricinus*, and only one presence and 88 absences for *I.* *persulcatus*. Collections were not conducted in 11 locations because of the tight collection schedule. A total of 502 ticks (35 adults, 375 nymphs, and 92 larvae) were collected from 26 locations and were pooled into 120 pools according to locality, species and development stage. Five hundred one ticks were identified as *I.* *ricinus* and only one nymph as *I.* *persulcatus*. We found no tick pools positive for TBEV. In contrast, 56 (47%) of the 120 tick pools were positive for *B.* *burgdorferi* (s.l.), consisting of 220 ticks, all identified as *I.* *ricinus*. The minimum infection rate (MIR) calculated for pooled ticks was 11.2%. Results from the additional collections in 2021 are shown in Additional file 1: Fig. S1b.

### Predictive model accuracies

We used four variable compositions for the predictions: environment only, host only, combined data on environmental and host variables, and combined data on environmental and host variables with suitability data of either tick species. The suitability data of *I.* *ricinus* and *I.* *persulcatus* for the previous variable composition were first produced using combined data of environmental and host variables.

The means and ranges of the predictive performances over 400 model runs by eight individual modelling techniques and four variable compositions are shown in Additional files 2 and 3: Figs. S2–S3. In all variable compositions, the GBM and RF models gained the highest model performances. The mean ensemble models were built over several modelling techniques, and the number of times they contributed to the final ensemble are seen in Additional file 4: Table S1. The mean ensembles estimating the habitat suitabilities yielded AUC values in the range of 0.90–0.94 (Table 2) for *I.* *ricinus* and 0.91–0.96 for *I.* *persulcatus*. The highest predictive performances from the four variable compositions were obtained with the combined data sets for both species in both the individual models and mean ensembles, and the lowest predictive performances were obtained with environmental data only (Additional files 2 and 3: Figs. S2–S3; Table 2).

**Table 2:** The predictive accuracy of the mean ensemble models in different variable compositions

	AUC	Sensitivity	Specificity
*I.* *ricinus*			
Environmental	0.90	78.3	84.2
Host	0.90	76	86.8
Environmental + Host	0.91	75.2	89.5
Environmental + Host + Habitat suitability for *I.* *persulcatus*	0.94	92.2	79.2
*I.* *persulcatus*			
Environmental	0.91	89.3	78.1
Host	0.92	91.4	78.7
Environmental + Host	0.93	92.7	78.3
Environmental + Host + Habitat suitability for *I.* *ricinus*	0.96	91.9	83.8

Sensitivity and specificity (by AUC) for estimating the distributions of *I.* *ricinus* and *I.* *persulcatus* varied based on the variable compositions. To estimate *I.* *ricinus* distributions, the mean ensemble model better identified unsuitable environments in all variable compositions (sensitivity = 75.2–78.3%, specificity = 84.2–89.5%), excluding the variable composition with environmental, host, and habitat suitability data of *I.* *persulcatus* (sensitivity = 92.2%, specificity = 79.2%). In contrast, when estimating *I.* *persulcatus* distributions, we found a better ability to identify suitable environments (sensitivity 89.3–92.7%) than unsuitable environments (specificity 78.1–83.8%). The highest sensitivity and specificity rates were obtained from the variable composition that contained all the variables for *I.* *persulcatus* (sensitivity = 91.9%, specificity = 83.8%).

### Predictor contributions to tick species distributions

Variable importance, which indicates the influence of the variable to the mean ensemble model, is referred to here as the relative contribution of the predictor (%). The higher the value, the more influence the variable has on the model. The relative contributions varied between the species and the variable compositions. Here, we present the relative contributions of the predictors used in the combined data sets (Fig. 3a, b), which gained the highest model performances. The relative contributions of the environmental only and host only data sets are shown in Additional file 5: Fig. S4a–b.

The highest relative contributions for *I.* *ricinus* predictions based on combined host and environmental data were obtained from the mean temperature of the *I.* *ricinus* activity season (42%), precipitation sum (16%), and red fox (15%), white-tailed deer (6%), and roe deer (4%) densities. For *I.* *persulcatus*, red fox density (24%), precipitation sum (23%), the densities of mountain hare (13%), white-tailed deer (10%), and European hare (9%), the mean precipitation for the *I.* *persulcatus* (5%) activity season, and roe deer density (4%) were the variables with the highest relative contributions. Predictions based on host, environmental, and habitat suitability data of the other species (Fig. 3b) indicated slightly different relative contributions. When predicting *I.* *ricinus* distributions, the highest relative contributions were obtained from the habitat suitability for *I.* *persulcatus* (74%), the mean temperature of the *I.* *ricinus* activity season (6%), and the red fox (5%), mountain hare (4%), and white-tailed deer (3%) densities. In contrast, the habitat suitability for *I.* *ricinus* (80%), night land surface temperature (6%), elevation (3%), and roe deer density (3%) were the most important predictors for *I.* *persulcatus*.

**Fig. 4:**
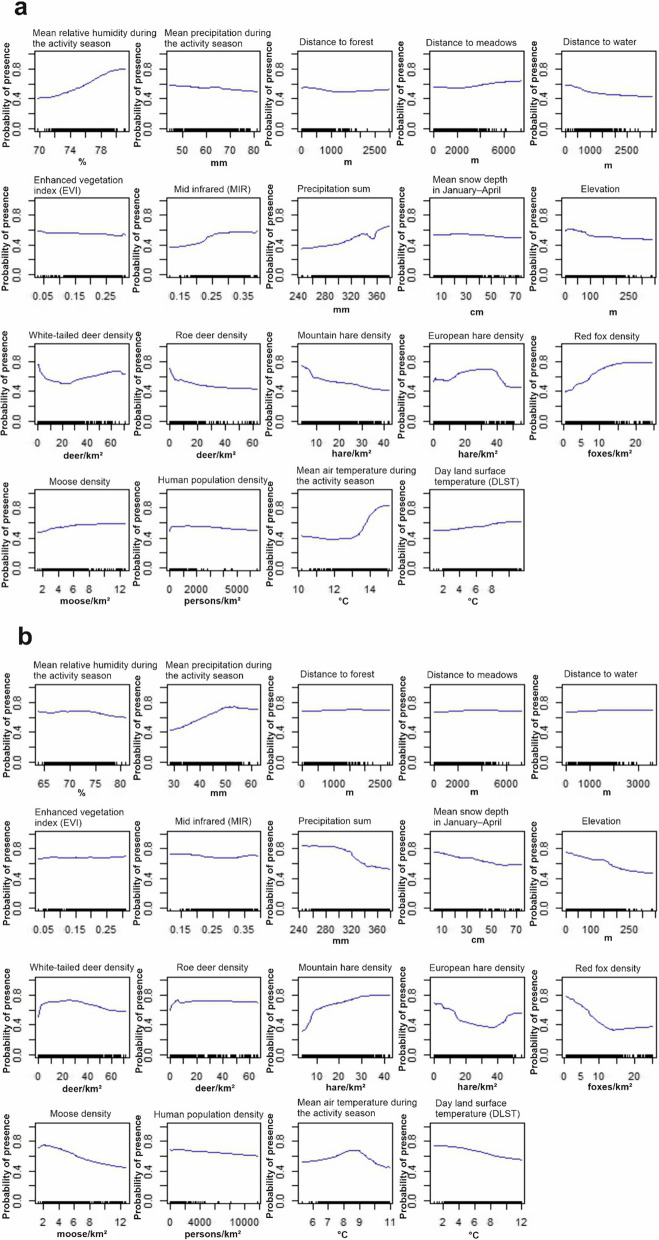
Partial dependency plots for **a** *I.* *ricinus* and **b** *I.* *persulcatus* based on combined host and environmental data produced by the mean ensemble model

We analysed partial dependency plots for *I.* *ricinus* and *I.* *persulcatus* based on the combined data set of environmental and host data (Fig. 4) because of the high influence of other species in the combined data set of environmental, host, and suitability data for the other species (see Fig. 3b). Partial dependency plots based on environmental only data, host only data, and a combined data set of environmental, host, and suitability data for the other species are shown in Additional file 6: Figs. S5–S7. Locations with higher relative humidity (> 75%), mean air temperature during the activity season (> 13 °C), precipitation sum (> 300 mm), middle-infrared reflectance (MIR, > 0.25), and higher white-tailed deer (> 30 individuals/km^2^), European hare (10–40/km^2^), and red fox (> 10/km^2^) densities were associated with higher habitat suitability for *I.* *ricinus* (Fig. 4a). However, higher mountain hare density (> 10/km^2^) was negatively associated with *I.* *ricinus* suitability. For *I.* *persulcatus*, higher mean precipitation (> 40 mm) and white-tailed deer (> 5/km^2^), roe deer (> 5/km^2^), and mountain hare (> 10/km^2^) densities were associated with high suitabilities for *I.* *persulcatus* (Fig. 4b). However, an excessively high precipitation sum (> 320 mm), higher mean air temperature during the activity season (> 9 °C), and day land surface temperature (DLST, > 10 °C) at the locations began negatively influencing the suitability for *I.* *persulcatus*. Furthermore, higher moose (> 8/km^2^) and red fox (> 10/km^2^) densities indicated lower suitabilities for *I.* *persulcatus* in the locations.

### Habitat suitabilities for *I. ricinus* and *I. persulcatus* based on variable compositions

**Fig. 5:**
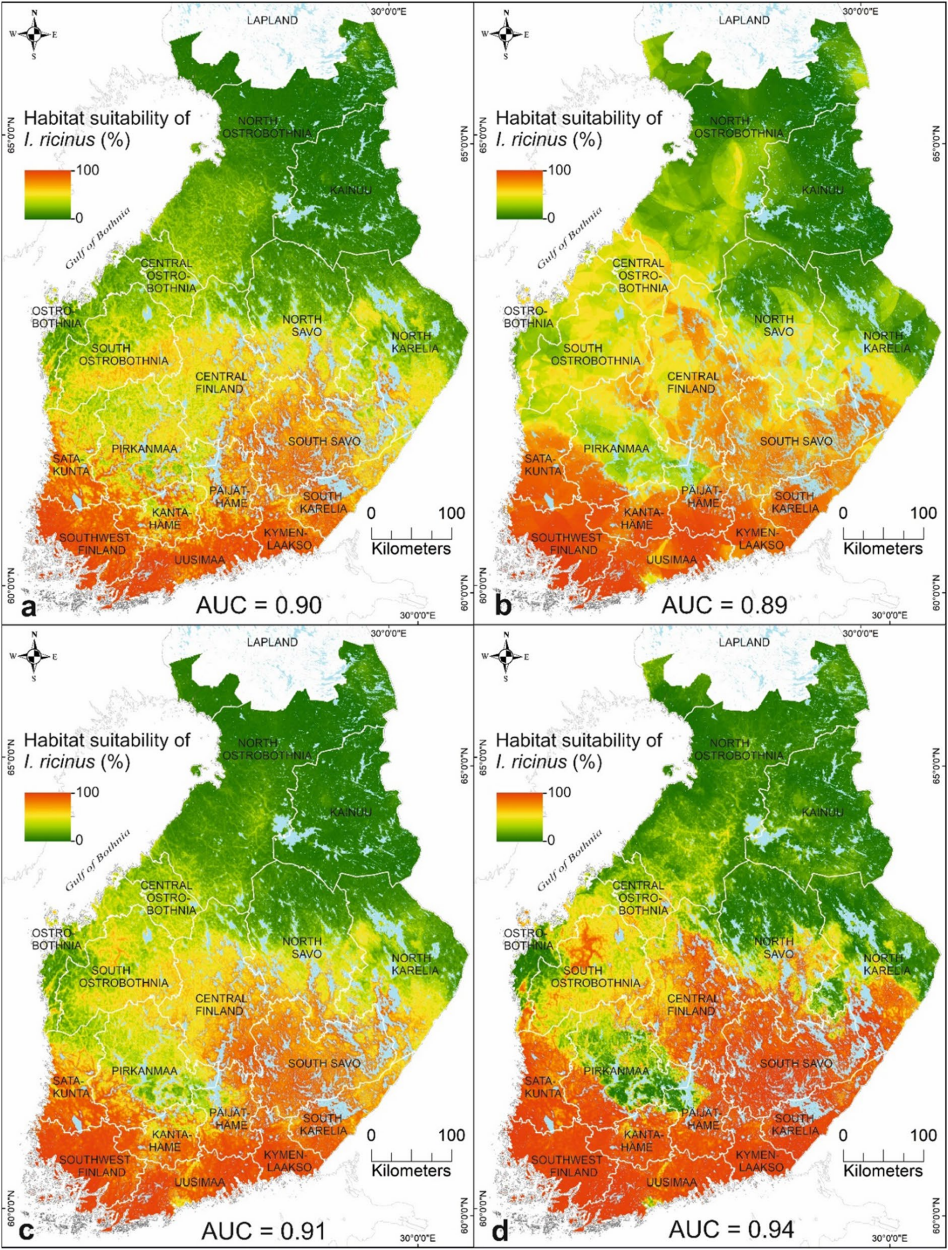
Estimated habitat suitabilities for *I.* *ricinus* in mainland Finland by the ensemble mean method over several modelling methods based on **a** environment only data, **b** host only data, **c** combined environmental and host data, and **d** combined environmental, host and, habitat suitability data for *I.* *persulcatus*

**Fig. 6:**
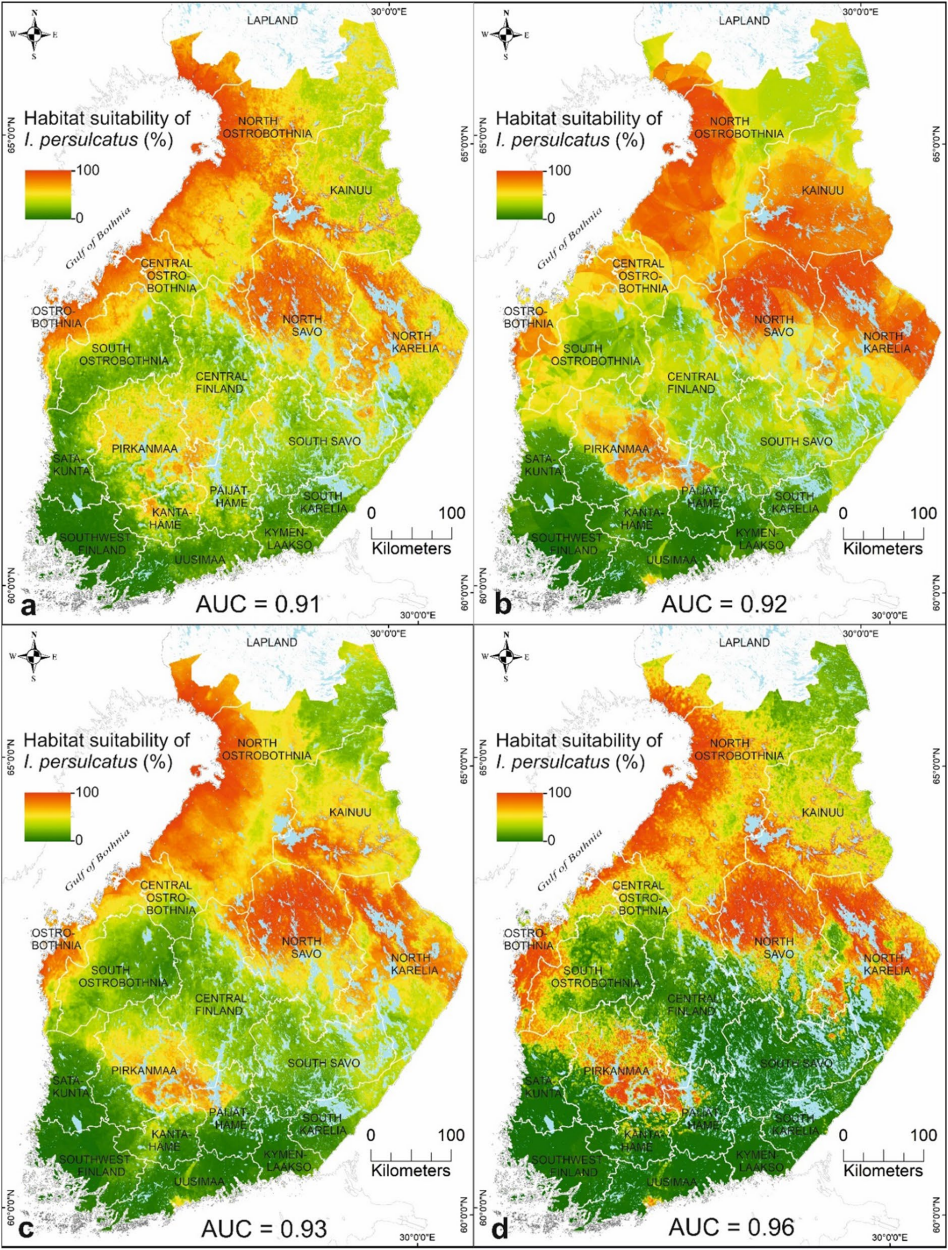
The estimated habitat suitabilities for *I.* *persulcatus* in mainland Finland by the ensemble mean method over several modelling methods based on **a** environment only data, **b** host only data, **c** combined environmental and host data, and **d** combined environmental, host, and habitat suitability data for *I.* *ricinus*

The habitat suitability maps for *I.* *ricinus* and *I.* *persulcatus* in four variable compositions are shown in Figs. 5 and 6. We focus on analysing the results from the combined data set of environmental and host data for *I.* *ricinus* (Fig. 5c) and *I.* *persulcatus* (Fig. 6c) based on the high predictive performance and the influence of the predictors contributing to the final ensemble model. In this study, low suitability for species presence is interpreted as 0–30%, moderate suitability as 31–60%, and high suitability as 61–100%. Figure 5c shows that the areas with moderate to high suitability for *I.* *ricinus* were located southwards from Central Ostrobothnia, with the following exceptions: narrow areas located in southern Pirkanmaa and southern coast of Ostrobothnia. Northern regions of North Savo, North Karelia, North Ostrobothnia, and Kainuu were estimated to have a low to moderate suitability for *I.* *ricinus*. Areas with moderate to high suitability for *I.* *persulcatus* were located mainly northwards from Ostrobothnia up to southern Lapland, including areas along the western coast, and eastern Finland (Fig. 6c). The moderate to high suitability areas for *I.* *persulcatus* in southern Finland were located across Pirkanmaa and in narrow areas of Kanta-Häme, Päijät-Häme, South Karelia, South Savo, and Uusimaa. Other areas in southern Finland, the northern part of Kainuu, and the eastern part of North Ostrobothnia were estimated to have a low to moderate probability for *I.* *persulcatus*.

## Discussion

### Study validity

No earlier SDM studies have estimated tick species distributions in Finland. However, there is a greater need for studying tick distributions when tick abundances and TBD incidences are increasing [80]. We used the mean ensemble model approach over several modelling methods, which are generally found to yield more accurate estimates than single-model estimates [81, 82] and are widely used to estimate the potential distributions of vectors and TBDs [83–85]. We assessed model performance using variable compositions and predictions uncertainties by using the coefficient of variation approach. Prediction uncertainties are often not considered in SDM studies, although the uncertainty assessment is highly recommended in SDM literature [86, 87]. This study obtained results at a 1-km resolution, which provides higher accuracy than generally in the SDM studies. The analyses utilized host data, which are often unavailable and thus are not included in a majority of tick SDM studies. Furthermore, species data were not aggregated from big data repositories, such as the Global Biodiversity Information Facility (GBIF), which are widely used but may affect results, e.g. due to spatial bias [88]. The results can be utilized as predictor data in future studies estimating the TBD risk in Finland.

### Predictive performance

Predictive performance of the mean ensembles over several modelling methods yielded model performances with minimum AUC values of 0.90 for both *I.* *ricinus* (Fig. 5) and *I.* *persulcatus* (Fig. 6). The mean ensemble models showed improvements of the AUC values compared with individual models (Additional files 2 and 3: Figs. S2–S3). Model performances can be classified into different categories: AUC values of 0.7–0.8 are considered fair model performance, 0.8–0.9 are good model performance, and > 0.9 are excellent model performance [89]. Mean ensembles yielded good to excellent model performances, while individual models resulted in fair to good model performances. Of the four variable compositions, ensemble models with combined data sets yielded the highest predictive accuracy for both *I.* *ricinus* (Fig. 5) and *I.* *persulcatus* (Fig. 6). Ecological and vegetation data, together with climatic data, are recommended for inclusion in the predictor data set when modelling species and disease distributions [86]. The ensemble model better identified unsuitable environments (i.e. specificity) in *I.* *ricinus* predictions in contrast to *I.* *persulcatus* predictions, in which suitable environments were better identified (i.e. sensitivity, Table 2). The high specificity rate for *I.* *ricinus* ensured that the proportion of true absences predicted as presences was minimized, and in contrast, the high sensitivity rate for *I.* *persulcatus* indicated that the proportion of true presences predicted as absences was minimized.

### The uncertainty assessment

Other influential factors are not included in this study such as microclimate and vertebrate density data. Vertebrate data, including e.g. rodent, bird, and raccoon dog densities, were not available in similar accuracy or spatial scale. Bank vole abundance in particular has previously been found to closely relate to questing *I.* *ricinus* abundances in Finland [12]. Along with rodents, birds are important hosts for *Ixodes* ticks [90]. However, bird density data covering the whole country are not available. Furthermore, some uncertainty arose from tick absences. The collections in 2021 resulted in 88 true absences for *I.* *persulcatus.* However, to obtain enough absences for *I.* *persulcatus*, we needed to use randomly selected points from the *I.* *ricinus* presences, obtained with the *Wallace* package. Only one thinned data set was obtained with *Wallace*, so we were unable to assess the occurrence-related uncertainty. True absences are difficult to obtain, and we are aware that the absences used in this study may not be true absences but may rather depend on e.g. collection time or weather conditions. As the ensemble model was better at identifying suitable than unsuitable environments for *I.* *persulcatus*, further studies are needed to bring new insights to whether this is an indication that the species is still spreading or whether this is due to modelling-specific reasons. Using presence-absence data instead of tick abundance data loses information on the relative suitability of habitats when all presences are treated as equal, regardless of the abundance of the individuals that the habitat supports. We excluded the Åland Islands and most of Lapland from the study due to the following reasons. The Åland Islands have abundant tick populations, and we did not have enough tick samples to reliably estimate their distributions. Only occasional tick observations have been made from Lapland, excluding the south-eastern municipalities of Kemi, Tornio, Keminmaa, and Simo.

**Fig. 7:**
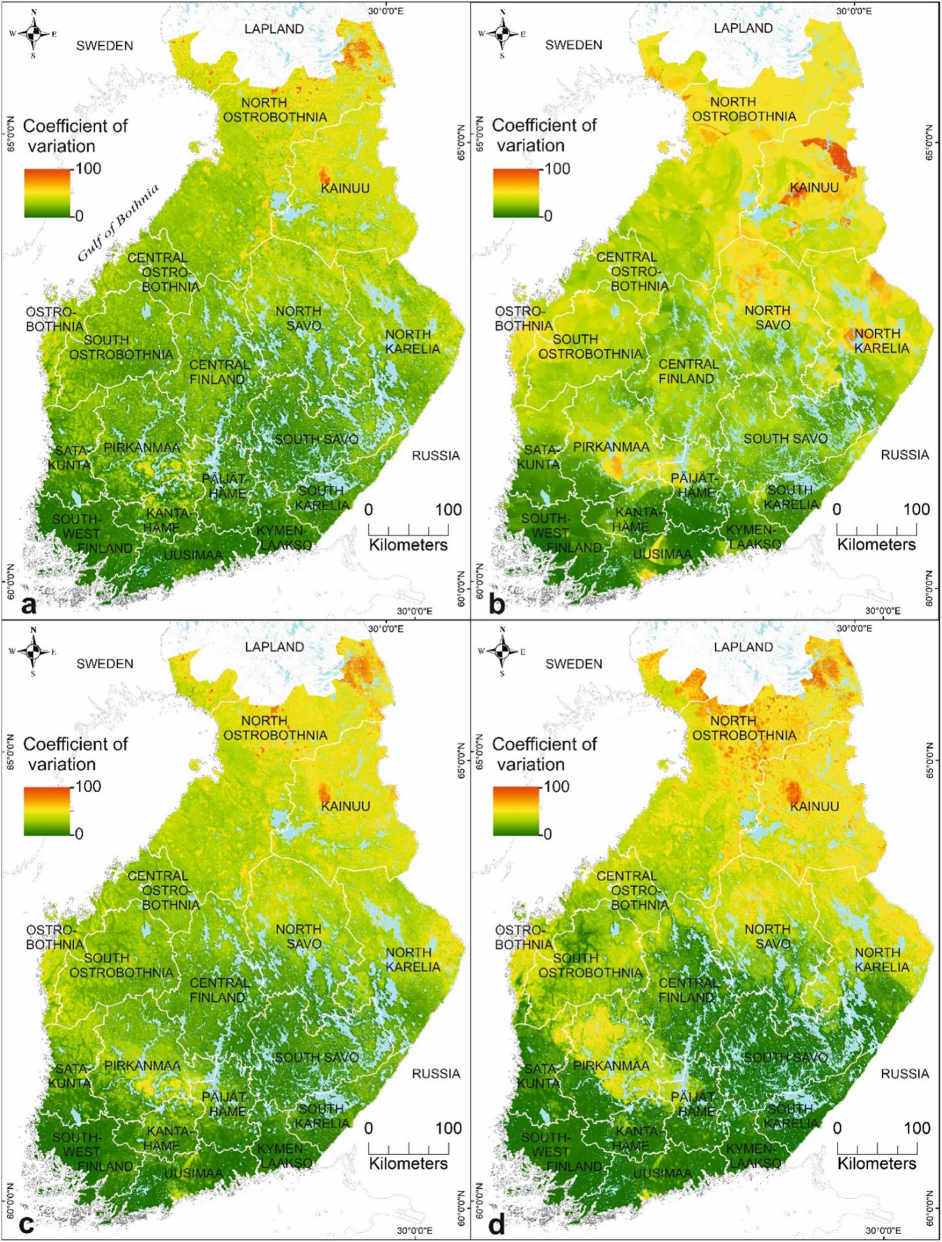
A coefficient of variation in the predictions estimating the uncertainty of the ensemble predictions over several modelling methods for *I.* *ricinus* in mainland Finland based on **a** environment only data, **b** host only data, **c** combined environmental and host data, and **d** combined environmental, host, and habitat suitability data for *I.* *persulcatus*

**Fig. 8:**
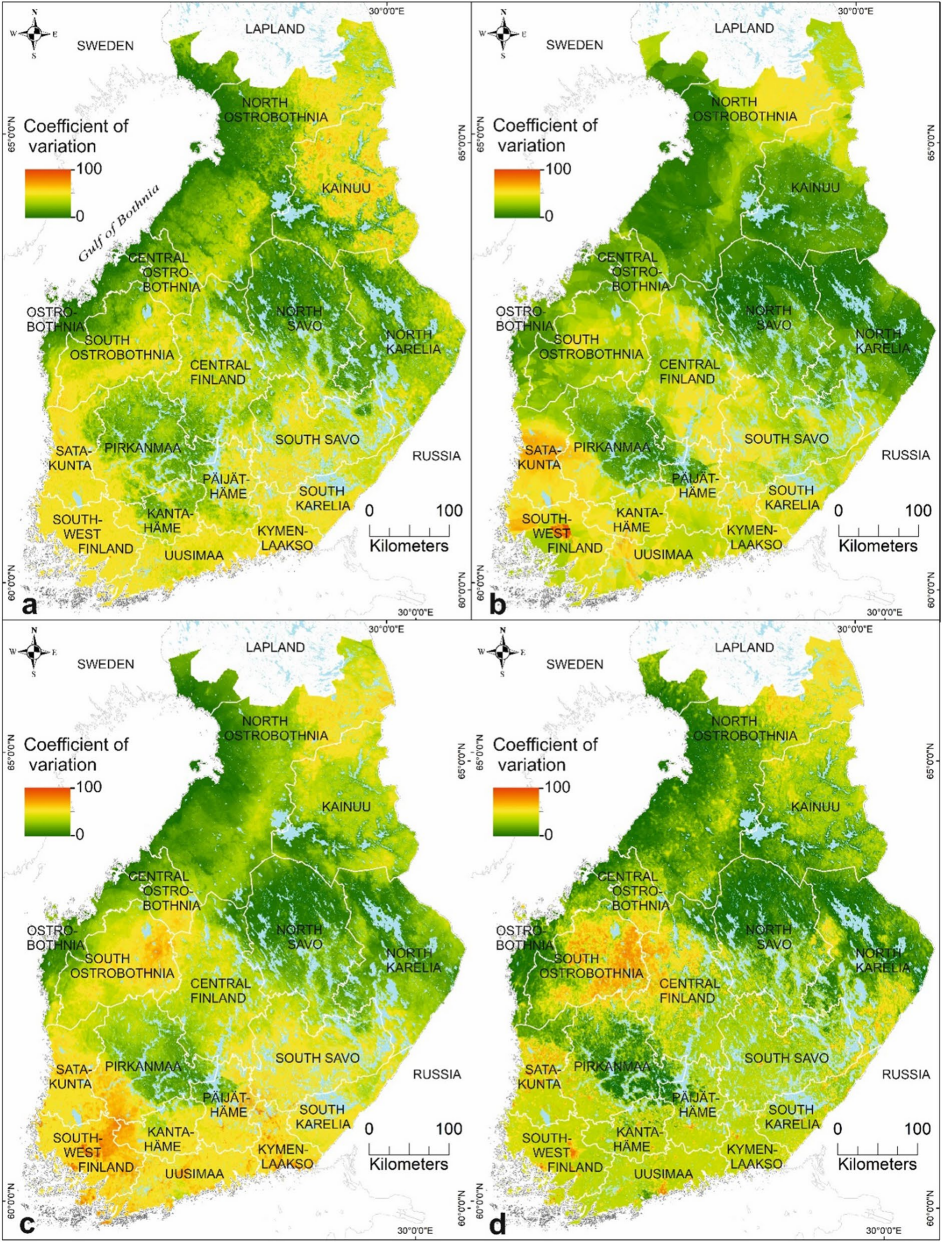
A coefficient of variation in the predictions assessing the uncertainty of the ensemble predictions over several modelling methods for *I.* *persulcatus* in mainland Finland based on **a** environment only data, **b** host only data, **c** combined environmental and host data, and **d** combined environmental, host, and habitat suitability data for *I.* *ricinus*

Hereafter, we focus on analysing the results based on the combined data set of environmental and host data due to high predictive performance (Figs. 5, 6), variable influences in the ensembles (Fig. 3a, b), and the recommendation to use ecological and vegetation factors with climatic factors in SDM [86]. The model-driven uncertainty of the predictions was presented using the coefficient of variation to assess the uncertainty of the suitability maps (Figs. 7, 8). In this study, low uncertainty is interpreted as 0–30%, moderate uncertainty as 31–60%, and high uncertainty as 61–100%. The areas with highest uncertainty for *I.* *ricinus* suitability were mainly located in North Ostrobothnia, southern Lapland, and Kainuu, and in narrow areas of Ostrobothnia, Pirkanmaa, and southern Uusimaa (Fig. 7c). High uncertainty in the northern and eastern parts of the study area may be associated with climatic factors of the areas characterized by shorter growing seasons, lower relative air humidity rates, and lower mean monthly air temperatures than in southern Finland. The lowest uncertainties were obtained from southern, eastern, central, and south-western Finland, indicating more reliable prediction results. For *I.* *persulcatus*, the areas with high uncertainty were somewhat larger in geographical extent than in the *I.* *ricinus* predictions (Fig. 8c). The highest prediction uncertainties were located across southern Finland, South Ostrobothnia, Central Finland, South Savo, Northern Kainuu, and north-eastern parts of North Ostrobothnia. In contrast, the areas with highest prediction confidence for *I.* *persulcatus* were located in coastal Finland northwards from Ostrobothnia up to southern Lapland, in Pirkanmaa, southern Kainuu, North Savo, and North Karelia.

### Influential factors

Consistent with previous research, the environmental, host, and climatic variables were important determinants for *I.* *ricinus* and *I.* *persulcatus* occurrence. Our study suggests that climatic factors, such as higher relative humidity, mean air temperature, and precipitation sum during the growing season, were associated with higher *I.* *ricinus* occurrence. Higher air temperatures [36, 37] and precipitation, especially in spring [91], have previously been found to positively influence *I.* *ricinus* presence. Based on our study results, higher red fox, white-tailed deer, and European hare densities were associated with higher habitat suitabilities for *I.* *ricinus*, which is mainly consistent with earlier findings (Fig. 3a) [92–95]. Although previous studies have found red foxes to be suitable hosts for ticks in Europe [96, 97], they were not considered suitable hosts in a recent study from Norway [93]. Mountain hare density had a negative effect on *I.* *ricinus* presence. This is obvious, as mountain hare densities have significantly declined in southern and western Finland during the past 30 years. However, as host densities may be directly and indirectly affected by climate, it is difficult to separate causal and confounding factors from one another [98, 99]. Consistent with previous research [36], our study suggests that high middle-infrared reflectance (MIR) levels positively influenced *I.* *ricinus* occurrence.

Similar to *I.* *ricinus*, *I.* *persulcatus* suitabilities were higher in locations with higher mean precipitation and air temperature during the activity season. However, when precipitation, mean air temperature, and daily land surface temperature (DLST) increased to a particular point, the suitability for *I.* *persulcatus* began decreasing. This finding may demonstrate the characteristics of *I.* *persulcatus* to prosper in slightly drier and colder habitats than *I.* *ricinus* [24]. However, the expansion of *I.* *persulcatus*' range has been found to correlate with the increase in mean annual air temperatures, which determine compatible temperature conditions for *I.* *persulcatus* establishment in new territories [100, 101]. As warmer winters and hotter summers are estimated to change the dynamics and pattern of seasonal tick activity [102], it remains to be seen whether *I.* *persulcatus* adapts to warmer and wetter habitat conditions. Some adaptation has already occurred with recent range expansion to southern parts of Finland [10]. In contrast to *I.* *ricinus*, higher red fox density negatively affected *I.* *persulcatus*. This is probably related to spatial variation in red fox density across the country; highest densities occur in southern parts decreasing steadily northward, to *I.* *persulcatus*-dominated area. Our study also suggests that higher densities of white-tailed deer, roe deer, and mountain hare were associated with higher habitat suitabilities for *I.* *persulcatus*, which is in line with previous findings [14, 99, 103]. Notably, white-tailed deer and roe deer populations have rapidly increased in southern Finland during the past few years, especially in the south-west [104], which may potentially affect not only the rise in *I.* *ricinus* abundances but also the southward spread of *I.* *persulcatus*.

### Habitat suitabilities for *I. ricinus* and *I. persulcatus* in Finland

*Ixodes* *ricinus* and *I.* *persulcatus* have their own environmental and other limits for surviving and reproducing, which restrict their geographical distributions. Based on our study results, moderate to high suitability areas for *I.* *ricinus* occurred throughout southern and Central Finland up to Central Ostrobothnia (64°N), excluding narrow areas in Ostrobothnia and Pirkanmaa (Fig. 6c). In neighbouring Sweden, only areas southwards from the capital region (60°N) were predicted to have abundant *I.* *ricinus* populations [36], despite *I.* *ricinus* having been found up to 66°N [99]. Based on recent *I.* *ricinus* studies from Russian Karelia, the species was absent already north of 63°N [105]. The narrow areas in Ostrobothnia and Pirkanmaa, considered sympatric areas, were estimated to have low suitability for *I.* *ricinus*, which may, partly, be explained by the moderate uncertainty in the prediction (Fig. 8c). The dominance area of *I.* *persulcatus* is known to be more northerly than that of *I.* *ricinus*. We note that the suitability for *I.* *persulcatus* was highest northwards from Ostrobothnia along the northern coast up to southern Lapland, Kainuu, North Savo, and North Karelia (Fig. 7c). Southern Finland, excluding areas in Pirkanmaa, western Päijät-Häme, northern Kanta-Häme, and southern Uusimaa, were estimated to have low habitat suitability for *I.* *persulcatus.* Also, there is a moderate to high uncertainty in the predictions for *I.* *persulcatus* across southern Finland (Fig. 8c), which may indicate that more areas with high suitabilities for the taiga tick may exist. Notably, *I.* *persulcatus* have only been found up to 63°N in Russian Karelia [43, 106]. In Sweden, the species was first observed in 2015 close to the Finnish border at ≈ 66°N [8].

## Conclusions

An increased risk of vector-borne pathogens and the spread of invasive and naturally spreading species due to changing weather patterns adds to the needs and requirements for increased research and concrete actions. In our study, habitat suitability areas for *I.* *ricinus* and *I.* *persulcatus* were identified for the first time in Finland. From additional tick collections in 2021, 25 new presences and 63 absences were found for *I.* *ricinus,* and 1 presence and 88 absences for *I.* *persulcatus*. A total of 502 ticks were analysed for pathogens, with no ticks positive for TBEV and ≈ 47% of tick pools positive for *Borrelia* *burgdorferi* (s.l.). High suitability areas for *I.* *ricinus* occurred throughout southern and Central Finland up to Central Ostrobothnia, excluding narrow areas in Ostrobothnia and Pirkanmaa. For *I.* *persulcatus*, the regions northwards from Ostrobothnia along the northern coast up to southern Lapland, Kainuu, North Savo, North Karelia, and areas in Pirkanmaa and Päijät-Häme were estimated to be suitable areas. Based on the predictions, locations with higher air temperature, relative humidity, precipitation sum, and MIR and higher densities of white-tailed deer, European hare, and red fox were suitable for *I.* *ricinus*. For *I.* *persulcatus*, higher mean precipitation and higher densities of white-tailed deer, roe deer, and mountain hare indicated a higher occurrence probability. The data produced in this study have implications for improving knowledge on disease prevention and for assisting authorities in decision-making concerning vector control strategies. Our results can be used as predictor data to estimate the risk for TBDs in Finland. In future studies, our aim is to focus on studying tick distributions at various spatial scales: in microhabitats and at larger scales covering all of Scandinavia. Our aims are to use in situ measurements to achieve more accurate microclimate data, to conduct more extensive tick sampling to enable tick abundance modelling, to model the distribution of TBPs in ticks, and to forecast tick distributions in the future climate.

## Supplementary Information

**Additional file 1: Figure S1.** (a) The sampling strategy for new collections in 2021 was created based on the following criteria. Subdivisions of landscape areas (Area1–Area4), CORINE land cover 2018, a 5-km buffer around existing *I. persulcatus* occurrences (grey circles), and a 500-m buffer around roads were used to delimit the four sampling areas (light grey lines). For each sampling area, a random sample of 25 collection locations was created depending on the relative shares of forest and meadow categories in each area. (b) The map showing the 2021 results indicates the locations where *I. ricinus* was found with B. burgdorferi (s.l.)-positive locations.

**Additional file 2: Figure S2.** The range (lines) and mean (dots) of model performances over 50 model runs in each model algorithm estimating habitat suitabilities for *I. ricinus* in different variable compositions: (a) environmental only, (b) host only, (c) environmental and host, and (d) environmental, host, and suitability for *I. ricinus*.

**Additional file 3: Figure S3.** The range (lines) and mean (dots) of model performances over 50 model runs in each model algorithm estimating habitat suitabilities for *I. persulcatus* in different variable compositions: (a) environmental only, (b) host only, (c) environmental and host, and (d) environmental, host, and suitability for *I. ricinus*.

**Additional file 4: Figure S4.** The relative contributions of the explanatory variables in the data set of (a) host only, (b) environment only based on the mean ensemble model.

**Additional file 5: Table S1.** The number of times each model contributed to the final ensemble in different data sets.

**Additional file 6: Figure S5.** Partial dependency plots for (a) *I. ricinus* and (b) *I. persulcatus* solely based on environmental data.

**Additional file 7: Figure S6.** Partial dependency plots for (a) *I. ricinus* and (b) *I. persulcatus* solely based on host data.

**Additional file 8: Figure S7.** Partial dependency plots for (a) *I. ricinus* and (b) *I. persulcatus* based on combined host and environmental data, and habitat suitability data for the other tick species.

## Data Availability

Data supporting the conclusions of this article are included within the article and its additional files. Raw data are available from the corresponding author upon request.

## Abbreviations

AUC: Area under the ROC curve
LB: Lyme borreliosis
MIR: Middle-infrared reflectance
PCR: Polymerase chain reaction
SDM: Species distribution modelling
TBD: Tick-borne disease
TBE: Tick-borne encephalitis
TBEV: Tick-borne encephalitis virus
TBP: Tick-borne pathogen
VIF: Variance inflation factor
