# Supplementary material for: Predicting habitat suitability for Ixodes ricinus and Ixodes persulcatus ticks in Finland
Source: Parasit Vectors. 2022 Aug 30;15:310. doi: 10.1186/s13071-022-05410-8 (PMC9429443; doi:10.1186/s13071-022-05410-8)

**Additional File 1: Figure S1.** **a** The sampling strategy for new collections in 2021 was created based on the following criteria. Subdivisions of landscape areas (Area1–Area4), CORINE land cover 2018, a 5-km buffer around existing *I. persulcatus* occurrences (grey circles), and a 500-m buffer around roads were used to delimit the four sampling areas (light grey lines). For each sampling area, a random sample of 25 collection locations were created depending on the relative shares of forest and meadow categories in each area. **b** The map showing the 2021 results indicates the locations where *I. ricinus* was found with *B. burgdorferi* s.l. positive locations.


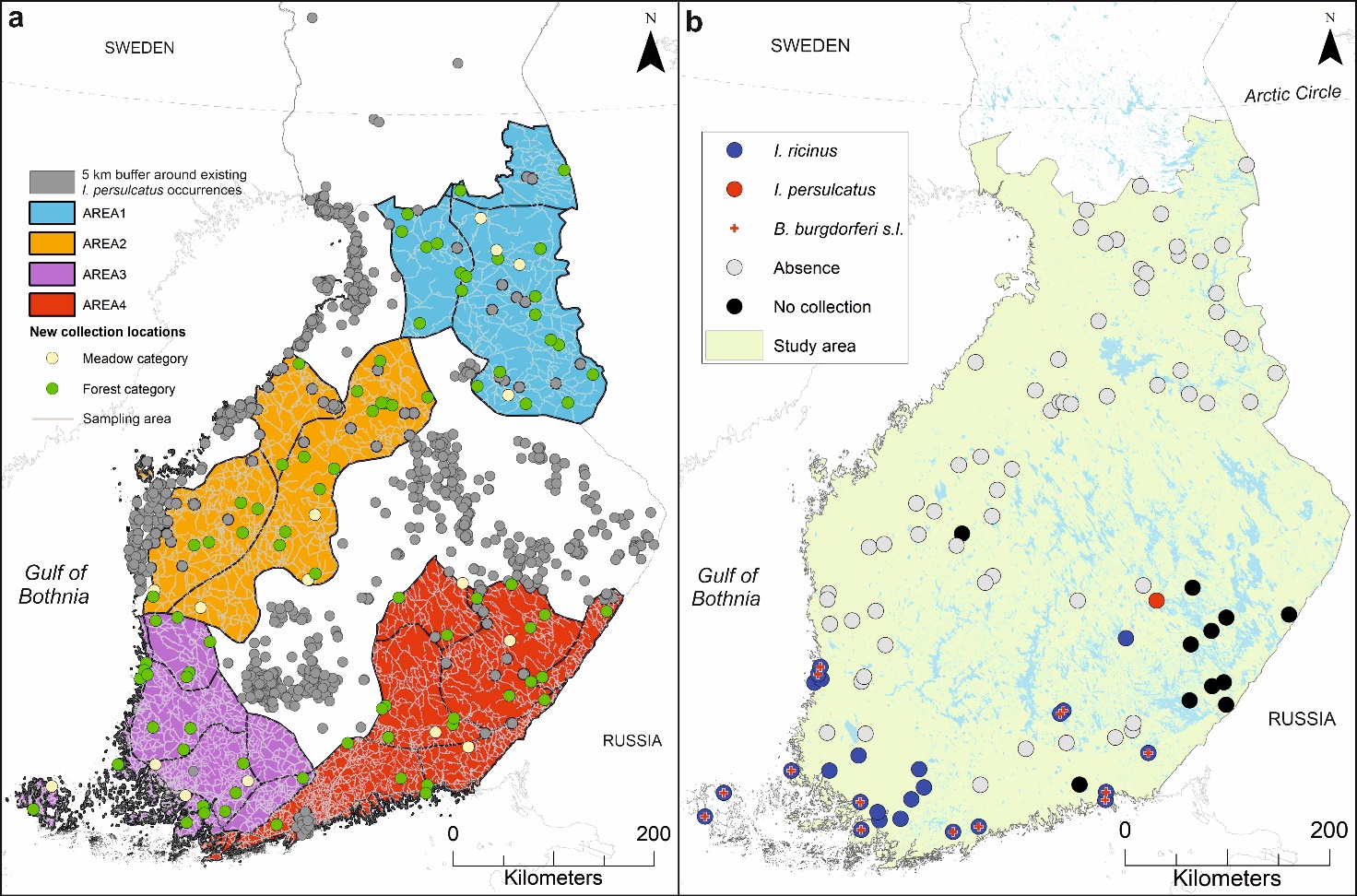

Supplement: Supplementary file 1 — Additional file 1: Figure S1. (a) The sampling strategy for new collections in 2021 was created based on the following criteria. Subdivisions of landscape areas (Area1–Area4), CORINE land cover 2018, a 5-km buffer around existing I. persulcatus occurrences (grey circles), and a 500-m buffer around roads were used to delimit the four sampling areas (light grey lines). For each sampling area, a random sample of 25 collection locations was created depending on the relative shares of forest and meadow categories in each area. (b) The map showing the 2021 results indicates the locations where I. ricinus was found with B. burgdorferi (s.l.)-positive locations. [file 13071_2022_5410_MOESM1_ESM.docx]
