# Supplementary material for: Predicting habitat suitability for Ixodes ricinus and Ixodes persulcatus ticks in Finland
Source: Parasit Vectors. 2022 Aug 30;15:310. doi: 10.1186/s13071-022-05410-8 (PMC9429443; doi:10.1186/s13071-022-05410-8)

**Additional File 3: Figure S3.** The range (lines) and mean (dots) of model performances over 50 model runs in each model algorithm estimating habitat suitabilities for *I. persulcatus* in different variable compositions: **a** environmental only, **b** host only, **c** environmental and host, and **d** environmental, host, and suitability for *I. ricinus*.


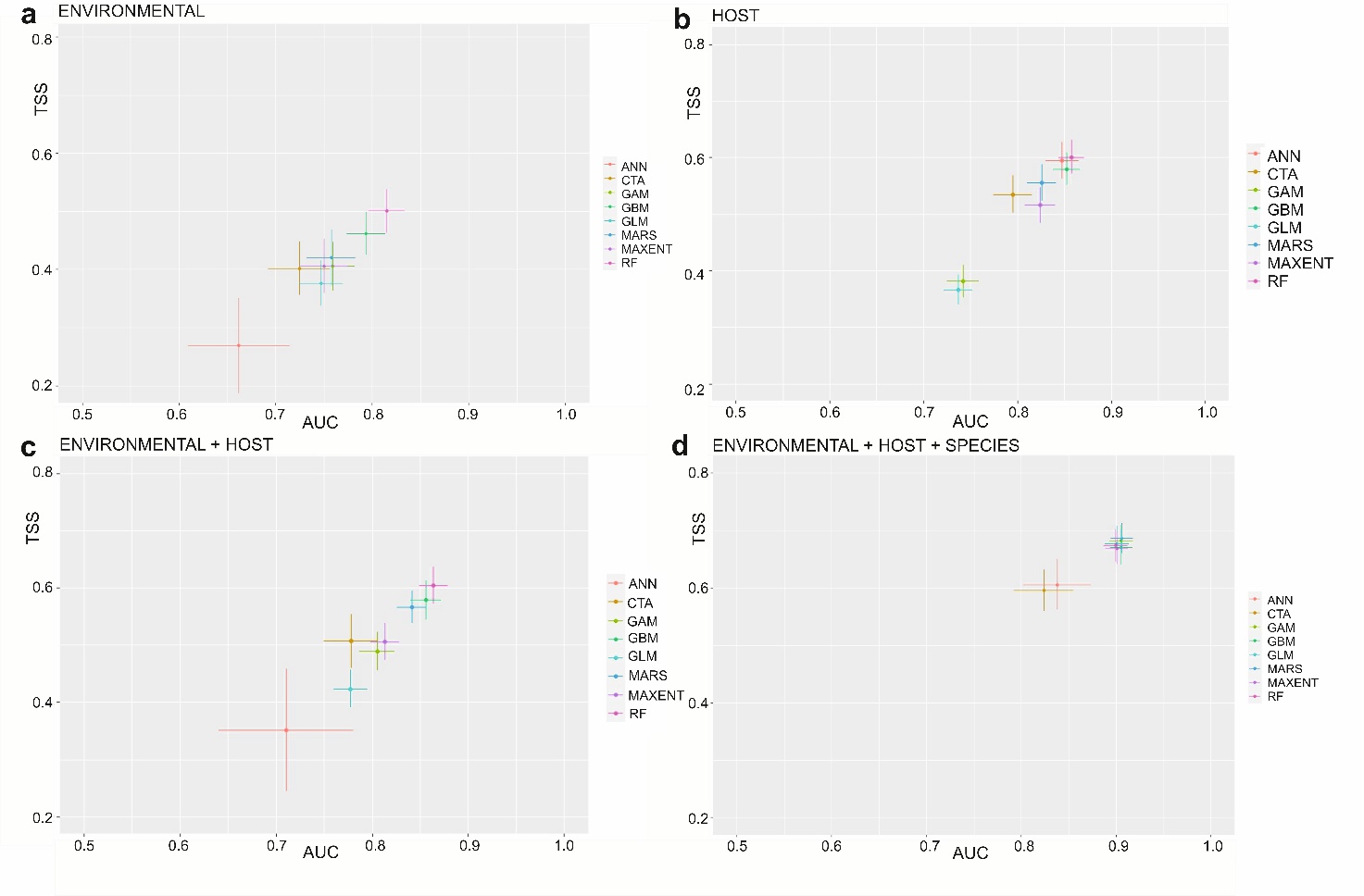

Supplement: Supplementary file 3 — Additional file 3: Figure S3. The range (lines) and mean (dots) of model performances over 50 model runs in each model algorithm estimating habitat suitabilities for I. persulcatus in different variable compositions: (a) environmental only, (b) host only, (c) environmental and host, and (d) environmental, host, and suitability for I. ricinus. [file 13071_2022_5410_MOESM3_ESM.docx]
