# Supplementary material for: Predicting habitat suitability for Ixodes ricinus and Ixodes persulcatus ticks in Finland
Source: Parasit Vectors. 2022 Aug 30;15:310. doi: 10.1186/s13071-022-05410-8 (PMC9429443; doi:10.1186/s13071-022-05410-8)

**Additional File 4: Figure S4.** The relative contributions of the explanatory variables in the data set of **a** host only, **b** environment only based on the mean ensemble model.


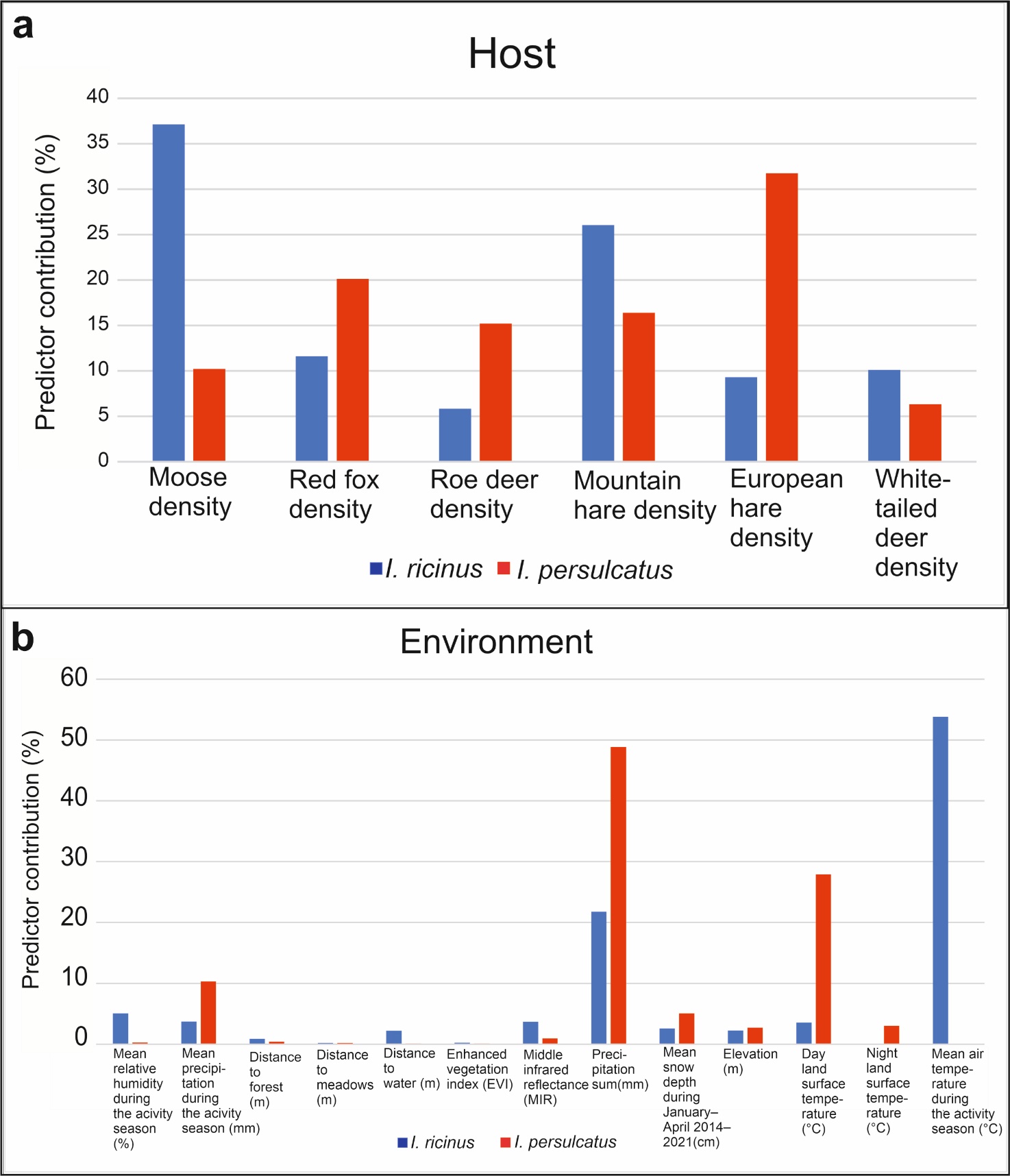

Supplement: Supplementary file 4 — Additional file 4: Figure S4. The relative contributions of the explanatory variables in the data set of (a) host only, (b) environment only based on the mean ensemble model. [file 13071_2022_5410_MOESM4_ESM.docx]
