# Supplementary material for: Predicting habitat suitability for Ixodes ricinus and Ixodes persulcatus ticks in Finland
Source: Parasit Vectors. 2022 Aug 30;15:310. doi: 10.1186/s13071-022-05410-8 (PMC9429443; doi:10.1186/s13071-022-05410-8)
Supplement: Supplementary file 5 — Additional file 5: Table S1. The number of times each model contributed to the final ensemble in different data sets. [file 13071_2022_5410_MOESM5_ESM.docx]

**Additional File 5: Table S1.** The number of times each model contributed to the final ensemble in different data sets.

| *I. ricinus* |  |  |  |  |
| --- | --- | --- | --- | --- |
|  | Environmental | Host | Environmental+Host | Environmental+Host+Species |
| GLM | 50 | 36 | 50 | 50 |
| GBM | 50 | 50 | 50 | 50 |
| GAM | 50 | 41 | 50 | 50 |
| CTA | 40 | 49 | 43 | 50 |
| ANN | 13 | 48 | 9 | 49 |
| MARS | 50 | 50 | 50 | 50 |
| RF | 50 | 50 | 50 | 50 |
| MAXENT | 49 | 50 | 50 | 50 |
|  |  |  |  |  |
| *I. persulcatus* | |  |  |  |
|  | Environmental | Host | Environmental+Host | Environmental+Host+Species |
| GLM | 36 | 49 | 50 | 50 |
| GBM | 50 | 50 | 50 | 50 |
| GAM | 41 | 50 | 50 | 50 |
| CTA | 49 | 50 | 50 | 50 |
| ANN | 50 | 50 | 38 | 50 |
| MARS | 50 | 50 | 50 | 50 |
| RF | 50 | 50 | 50 | 50 |
| MAXENT | 50 | 50 | 50 | 50 |
