# Supplementary material for: Predicting habitat suitability for Ixodes ricinus and Ixodes persulcatus ticks in Finland
Source: Parasit Vectors. 2022 Aug 30;15:310. doi: 10.1186/s13071-022-05410-8 (PMC9429443; doi:10.1186/s13071-022-05410-8)

**Additional File 6: Figure S5.** Partial dependency plots for **a** *I. ricinus* and **b** *I. persulcatus* solely based on environmental data.


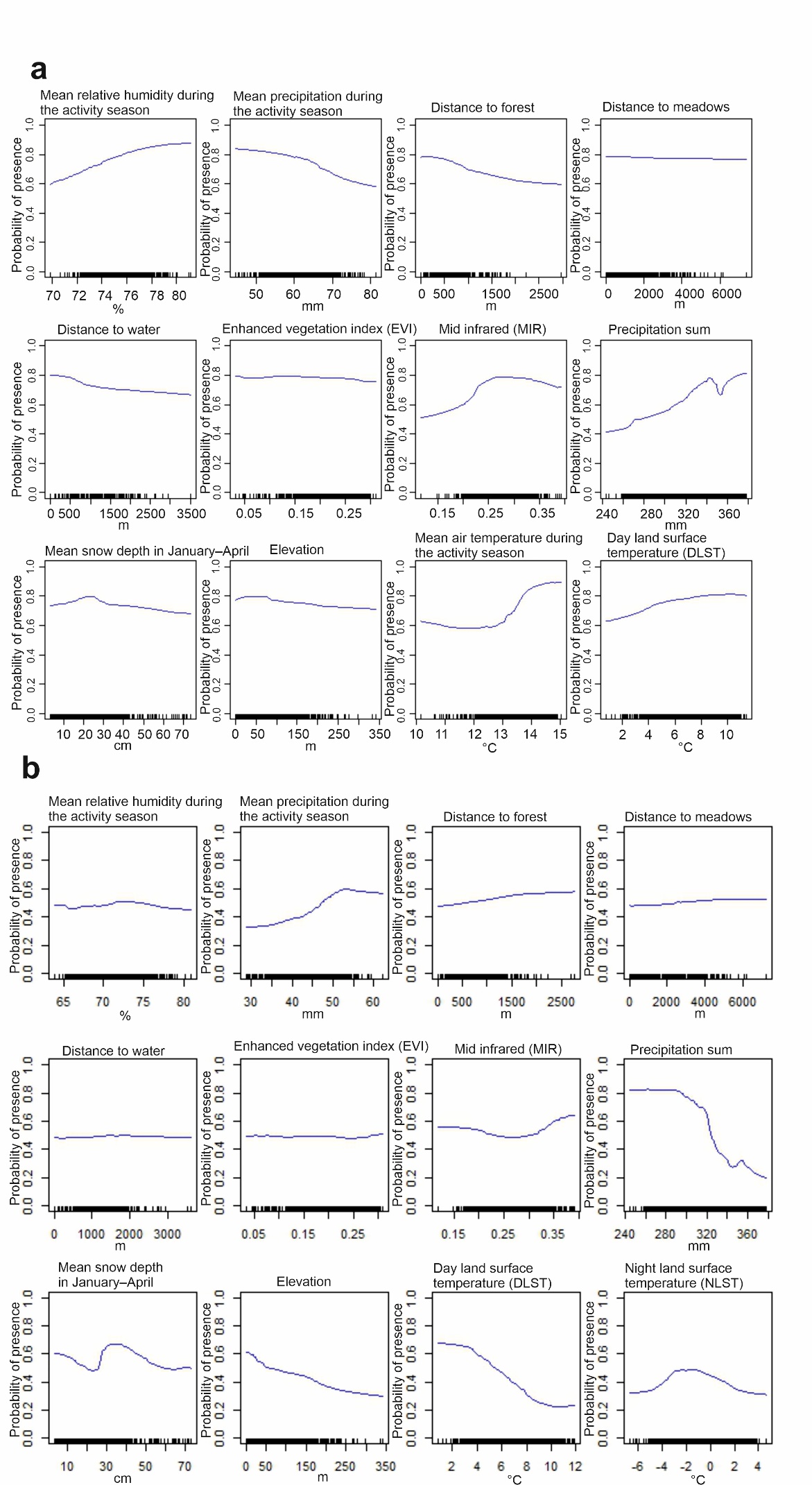

Supplement: Supplementary file 6 — Additional file 6: Figure S5. Partial dependency plots for (a) I. ricinus and (b) I. persulcatus solely based on environmental data. [file 13071_2022_5410_MOESM6_ESM.docx]
